# Observation of a giant nonlinear wave-packet on the surface of the ocean

**DOI:** 10.1038/s41598-021-02875-y

**Published:** 2021-12-08

**Authors:** Miguel Onorato, Luigi Cavaleri, Stephane Randoux, Pierre Suret, Maria Isabel Ruiz, Marta de Alfonso, Alvise Benetazzo

**Affiliations:** 1 Dipartimento di Fisica, Università degli Studi di Torino, 10125 Turin, Italy; 2 Istituto Nazionale di Fisica Nucleare, INFN, Sezione di Torino, 10125 Turin, Italy; 3 Istituto di Scienze Marine (ISMAR), Consiglio Nazionale delle Ricerche (CNR), Venice, Italy; 4 Univ. Lille, CNRS, UMR 8523-PhLAM-Physique des Lasers Atomes et Molécules, 59000 Lille, France; 5 Puertos del Estado, Madrid, Spain

**Keywords:** Fluid dynamics, Physics, Statistical physics, thermodynamics and nonlinear dynamics, Nonlinear phenomena

## Abstract

In many physical systems such as ocean waves, nonlinear optics, plasma physics etc., extreme events and rare fluctuations of a wave field have been widely observed and discussed. In the field of oceanography and naval architecture, their understanding is fundamental for a correct design of platforms and ships, and for performing safe operations at sea. Here, we report a measurement of an impressive and unique wave packet recorded in the Bay of Biscay in the North-East of the Atlantic Ocean. An analysis of the spatial extension of the packet that includes three large waves reveals that it extents for more than 1 km, with individual crests moving faster than 100 km/h. The central and largest wave in the packet was 27.8 m high in a sea with significant wave height of 11 m. A detailed analysis of the data using the nonlinear Fourier analysis reveals that the wave packet is characterized by a non trivial nonlinear content. This observation opens a new paradigm which requires new understanding of the dynamics of ocean waves and, more in general, of nonlinear and dispersive waves.

## Introduction

Ocean surface gravity waves are fascinating perturbations of the interface between the atmosphere and the sea: their oscillating properties are a direct consequence of gravity that acts as the restoring force. Apart from tsunamis, whose origin is a fast displacement of a large volume of water related to a telluric movement, ocean waves are generated by winds that slowly, but inexorably, transfer them energy. Randomness is a peculiarity of ocean wind waves; indeed, very seldom the wave dynamics under the effect of wind can be considered as a coherent process. The reasons is not unique: first of all wind forcing is in general turbulent and characterized by random fluctuations; moreover, surface gravity waves are dispersive, which inevitably imposes a finite life time of any localised coherent wave packet. This mechanism holds true unless nonlinearity, which is another intrinsic feature of finite amplitude ocean waves, enters into the game, balancing the dispersion. Such combination of effects leads eventually to the formation of localised structures which are known as solitons, i.e localised traveling waves solutions of integrable partial differential equations, the latter derived from a drastic reduction of the primitive fluid mechanical equations of motion. The existence of envelope solitons or breathers in the real ocean has been debated in the past literature^1–3^; here we give evidence of the existence of a localized nonlinear structure on the sea surface which eventually will have a finite life time.

Most of the times, if a time series of the surface elevation is recorded, its Fourier spectrum is characterized by some width which is inversely proportional to the local dispersion time scale and by some independent Fourier phases. Those are the prerequisite for a Gaussian probability density function of the surface elevation which indeed, with small deviations, is the one that mostly characterizes ocean waves^4^. More rarely, in conditions that still needs to be understood, extreme events, known as rogue waves, may appear on the surface of the ocean^5–10^. While this phenomenon is now well documented in the oceanographic literature^11^, its origin is still very much debated^12^: either a rogue wave is a rare event belonging to a quasi-Gaussian distribution or it is a more frequent (in some sea states) event pertaining to a different population. What one usually observes from time series recorded in the ocean is a large crest embedded in a random sea state characterized by a significant wave height that is smaller than half the height of the largest wave. The average shape (over different rogue waves measured in similar sea state conditions) can be predicted^13–16^.

## Results

**Figure 1:**
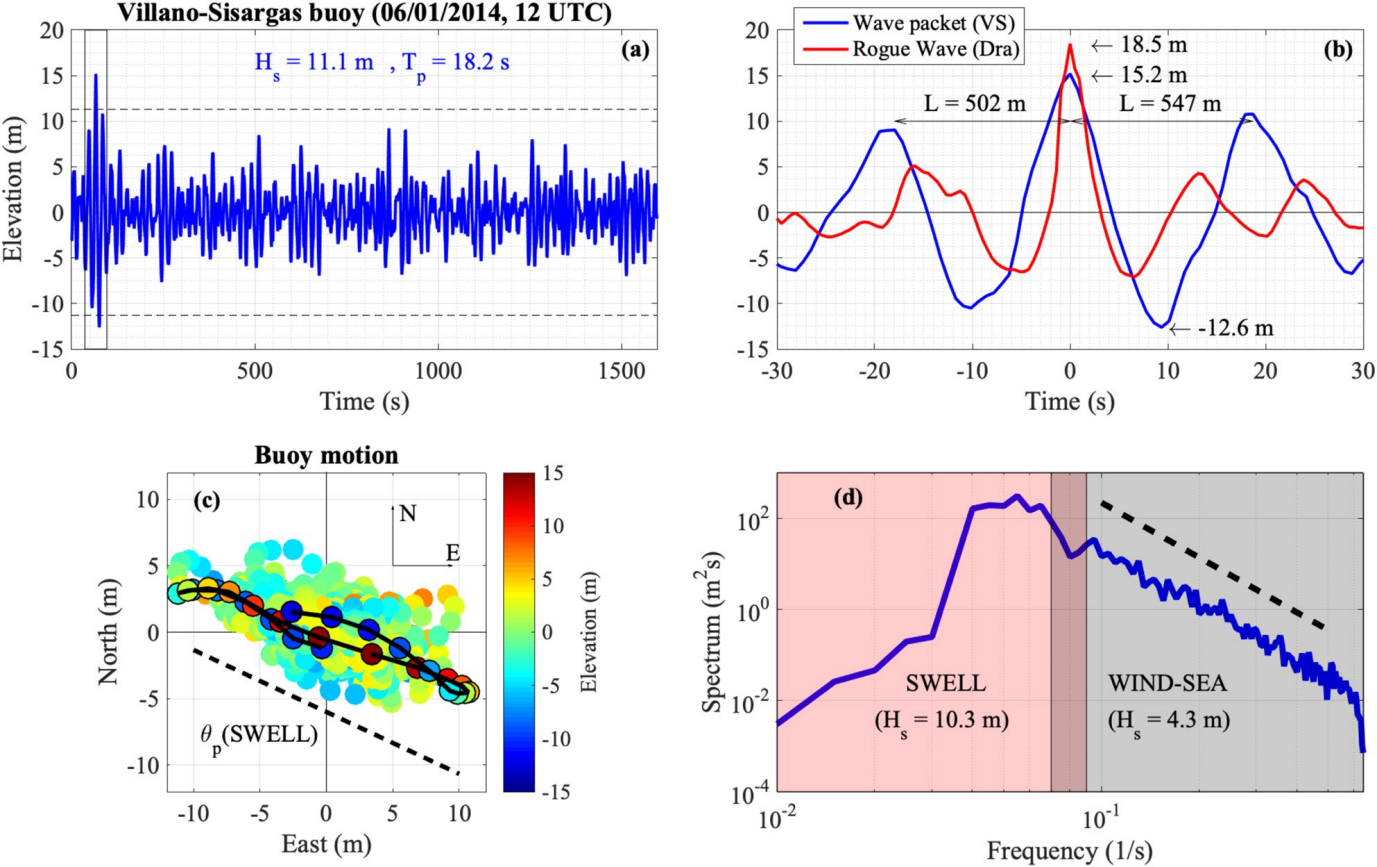
Measurement in the Gulf of Biscay. (**a**) The time series of sea surface elevation showing the giant wave packet. (**b**) A comparison of the famous Draupner rogue wave (red line) and the giant wave packet measured in the Gulf of Biscay (blue line). Time in the horizontal axis has been shifted in order to display the crest of the largest waves at time $t=0$. The distance L between the crests is obtained by means of the linear dispersion relation. Arrows indicate crest heights and trough depth. (**c**) East–North displacement of the buoy for the whole record (circles) and between the two troughs across the maximum elevation (edges circles). The marker colour is proportional to the surface elevation. (**d**) The frequency spectrum has been computed on the time series using standard FFT routine. Averages over samples of 1024 points ($\Delta$t = 0.78 s) with overlap of 50$\%$ have been performed. The logarithmic scale spectrum shows a clear peak at 0.054 Hz which corresponds to a period of 18.5 s^18^.

Here, we report on a related phenomenon, i.e. the existence of a giant wave packet on the ocean surface, i.e. a group of coherent waves that emerge from a random sea state, see Fig. 1a: the central wave in the packet, the largest one, has a height of almost 27.8 m. To highlight the difference between a typical rogue wave and the giant wave-packet in Fig. 1b, we show a zoom of the measurements in the Gulf of Biscay and the well known Draupner wave^12,17^, (a shift in time has been applied in order to have the crest of the largest waves both at time $t=0$): while the crest of the Draupner wave is slightly larger, the different size and volume of water involved in the two events is evident. Using the method described in^18^, the study of the meteocean conditions during which such object has been recorded and the understanding of its content in terms of the Nonlinear Fourier analysis are the subject of this article.

**Figure 2:**
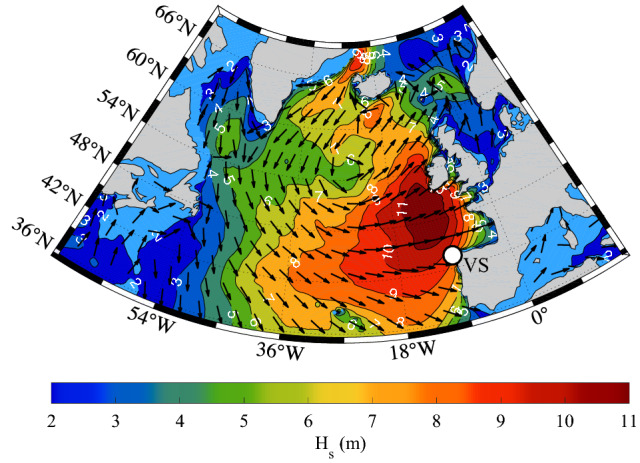
Wave conditions. Significant wave height (m) and mean wave direction (arrows, decimated for graphical purposes) on January 06, 2014, at 12 UTC. Note the large area of high energy propagating from the Atlantic Ocean towards the Iberian peninsula (the white dots shows the location of the Villano-Sisargas buoy, VS).

The winter 2013–2014 has been the most active one of the last decade in the North Atlantic, the 4–6 January 2014 storm representing the most severe event of the season. The storm initiated on 3 January with an intensifying low pressure system off Newfoundland. In the following day the storm grew very rapidly, surface pressure dropping at 934 hPa, satisfying the condition of explosive cyclogenesis. Moving rapidly East, the strong winds, measured up to 28 m/s, led to exceptionally large waves, the peak conditions (according to the analysis of the European Centre for Medium-Range Weather Forecasts, ECMWF) being close to 20 m significant wave height. While the heart of the storm moved towards Ireland where the largest coastal waves took place, the cyclonic winds led also to a strong south-easterly moving swell that reached the coasts of the Iberian peninsula on 6 January. Heavy damage was reported along the Portuguese coast. When the swell reached the north-west corner of the peninsula, a south-westerly wind was locally blowing (Fig. 2). At 12 UTC the buoy of Villano-Sisargas (Cabo Vilán), located at $43.50^\circ$N and $9.21^\circ$W, close to the north-west corner of the Iberian peninsula, where the water depth *h* is 383 m, recorded the giant wave packet. The local wave conditions were characterized by a dominant South-East directed swell plus a superimposed relatively minor wind sea directed to 60$^{\circ }$N. The mean directions plotted in the panels of Fig. 2 represent the weighted combination of the two respective energies.

**Figure 3:**
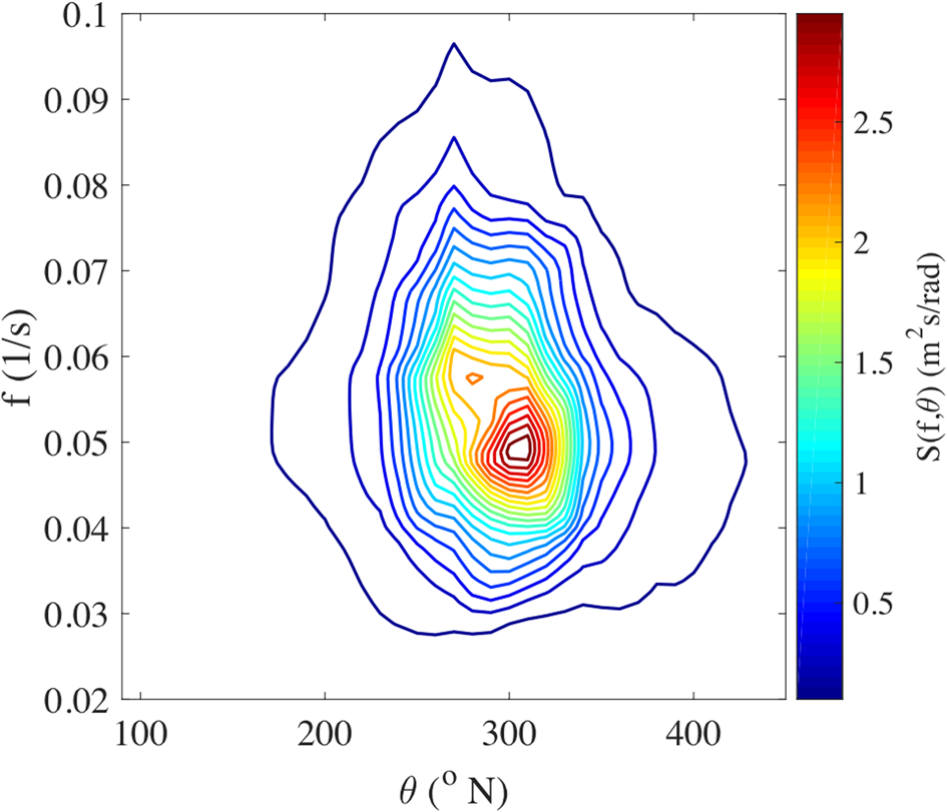
Directional spectrum computed using the Extended Maximum Likelihood Method. The largest peak at lower frequency corresponds to the swell propagating from North-West. The smaller peak is related to a wind sea in generation.

The largest wave measured is of 27.8 m in a significant wave height height, $H_s$, of 11.1 m, the latter calculated as 4 times the standard deviation of the 1600 s time series shown in Fig. 1 (sampling rate 1.28 Hz). According to the definition of a rogue waves, $H_{rogue}>2 H_s$, it qualifies as a rogue wave. Moreover, the successive wave in the packet satisfies the criterium $H>2 H_s$, its height being 23.4 m. This is an exceptional case in which two adjacent waves in a wave packet are both rogue (according to the mentioned criterion). Recently, a triplet of consecutive relatively large waves at Ekofisk, but on much lower wave conditions (4 m significant wave height) has been reported^19^. In the present case the distance (in time) between the two local minima around the largest wave is 19.5 s which, according to the linear dispersion relation, corresponds to a length of 593 m. Besides measuring the surface elevation, the position of the buoy in time has been recorded, its trajectory following, in deep water conditions, to some extent the local wave kinematics. In Fig. 1c the horizontal displacement of the buoy is shown. The colours in the plot indicate the vertical displacement; dark dots connected with a line indicate the time interval during which the large wave passed. For perfectly unidirectional waves, the motion of the buoy would be on a line inclined of some angle, depending on the direction of propagation of the waves. Interestingly, the Figure confirms that all waves in the wave packet travel in the same direction. The frequency-direction spectrum has been computed using the buoy 2-D horizontal displacement and heave implemented in the Extended Maximum Likelihood Method^18^, see Fig. 3. The spectrum displays two peaks with a small angle in between them. The higher frequency peak is related to the wind sea while the other more energetic peak corresponds to the swell.

## Methods

The results shown in Fig. 1c,d, the reconstruction of the storm in Fig. 2 and the energetic swell observable in the directional spectrum, allow us to assume that locally the dynamics can be approximated at least for short times to one dimensional propagation. Within this framework, a model that describes to the leading order in dispersion and nonlinearity the dynamics of surface gravity waves in infinite water depth as is the Nonlinear Schrödinger equation, written as an evolution equation in space, which reads:

1 $$\begin{aligned} i \frac{\partial A}{\partial z} + \frac{\kappa }{\nu ^3} \frac{k_0}{\omega _0^2} \frac{\partial ^2 A}{\partial t^2} + \frac{\gamma }{\nu } k_0^3 |A|^2 A=0, \end{aligned}$$

with

2 $$\begin{aligned} \nu &=1+ \frac{2k_0 h}{\sinh {(2k_0 h)}},\;\;\;\; \kappa =-\nu ^2+2+8(k_0h)^2 \frac{\cosh {(2k_0 h)}}{\sinh ^2{(2k_0 h)}},\\ \gamma &= \frac{\cosh {(4k_0 h)}+8-2\tanh ^2{(k_0h)}}{8\sinh ^4{(k_0 h)}} - \frac{(2\cosh ^2{(k_0 h)}+0.5\nu )^2}{\sinh ^2{(2k_0 h)}} \left( \frac{k_0h}{\tanh {(k_0h)}}-\frac{\nu ^2}{4} \right) \end{aligned}$$

$A=A(x, t)$ represents the complex envelope of the surface elevation that changes in space *x* and in time *t*. $k_0$ and $\omega _0$ are the wave number of the carrier wave and its associated frequency, respectively. They can be estimated directly from a time series taking its Fourier spectrum and considering the frequency corresponding to its peak. For what follows the angular frequency $\omega _0$ will be taken to be 0.314 s$^{-1}$ ($f_0=\omega _0/(2 \pi )=0.05$ Hz); with this value of the angular frequency, the wavenumber $k_0=\omega _0^2/g$ has the numerical value $k_0=10^{-2}$ $m^{-1}$ and $k_0 h=3.83$. The finite water depth has drastic effects on the dynamics: the NLS equation is still in a focussing regime, since $k_0 h>1.36$; the numerical values of the coefficients are $\nu =1.01$, $\gamma =0.72$, $\kappa =1.09$

The surface elevation, $\eta (x,t)$ is related to the complex envelope at leading order as follows:

3 $$\begin{aligned} \eta (x,t)=\frac{1}{2} \left( A e^{i (k_0 x-\omega _0 t)}+ A^* e^{-i (k_0 x-\omega _0 t)}\right) \end{aligned}$$

The NLS equation is integrable through the Inverse Scattering Transform^20^, IST, which can be considered as a generalization of the standard Fourier Transform which solves linear wave problems. Just like the Fourier analysis, the IST can be used to analyze data. Indeed, starting from the pioneering works in^21,22^, a nonlinear time series analysis tool has been developed and used for different applications^23–28^. Here we analyze the time series by solving the nonlinear spectral problem associated with Eq. (1):

4 $$\begin{aligned} \frac{d }{dt} \begin{pmatrix} \psi _1\\ \psi _2 \end{pmatrix} = \begin{pmatrix} -i \lambda &{}\quad A_0 \\ -A_0^* &{}\quad i \lambda \\ \end{pmatrix} \begin{pmatrix} \psi _1\\ \psi _2 \end{pmatrix} \end{aligned}$$

where $A_0=A_0(t)$ is the time series recorded; $\psi _1$ and $\psi _2$ are the eigenfunctions of the spectral problem (just like $\sin$ and $\cos$ are eigenfunctions in the linear Fourier analysis). $\lambda$s represent the complex eigenvalues (the equivalent of the frequencies or wave numbers); if the evolution of the data is based on the NLS equation, the $\lambda$s do not evolve (just like the absolute value of the Fourier coefficients do not evolve in time if the evolution is linear). Indeed, the main tool for visualizing the results from a nonlinear spectral analysis is the so called $\lambda$-plane. $\lambda$ can have a real and imaginary part, the latter forming the discrete spectrum associated with the soliton content of $A_0$. As prescribed in^24,25^, the field $A_0$ used in Eq. (4) is obtained by limiting the experimental signal over a time interval of finite extent in order to establish locally the nonlinear contents of the data. For each window, the spectral problem in Eq. (4) has been solved using zero (ZBC)^25^ and periodic boundary (PBC) conditions^22,29,30^

**Figure 4:**
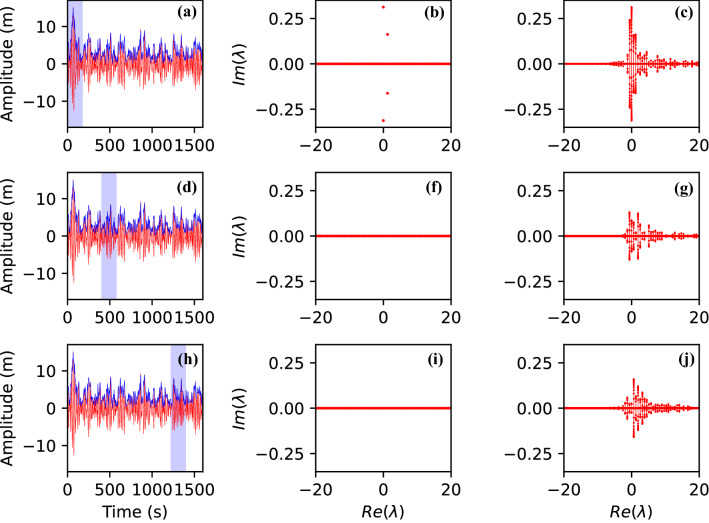
Left column: the signal recorded by the buoy is plotted in red line. The blue lines represent the modulus of the envelope computed by using Hilbert transform method. The blue shaded area indicates the region considered for the nonlinear spectral analysis. In the three cases reported in the left column, the width of the time window used for IST analysis is 180 s. Central column: discrete IST spectra computed using the experimental signal plotted in the blue shaded regions shown in the left column: zero boundary conditions (ZBC) are applied. Two discrete eigenvalues are found well above the real axis only for the large wave packet (see upper line). Right column: the spectra computed using the experimental signal plotted in the blue shaded regions shown in the left column: periodic boundary conditions (PBC) are applied. With these boundary conditions, spines crossing the real horizontal axis are obtained instead of discrete eigenvalues that are obtained with ZBC. Using PBC spines are spectral signatures of nonlinear modes. In (**c**) two spines of large amplitude are obtained when the giant wave packet is analyzed. Those two spines are not found when the window of analysis does not overlap the giant wavepacket.

In Fig. 4 we show the results of our analysis by plotting on the left the time series (the window of time over which the analysis has been performed is shaded) and on the central and right columns the nonlinear analysis performed with ZBC and PBC, respectively. Using ZBC, two discrete eigenvalues located above the real axis are obtained when the analysis is performed on a window that contains the large wave packet. These discrete eigenvalues are not found if the nonlinear spectral analysis is performed at other positions. The analysis has also been performed on wider windows confirming the same results. Similar results are obtained if PBC are used: instead of discrete eigenvalues, two large spines are observed in correspondence of the large wave packet. Those spines are a signature of nonlinear modes that can be interpreted in the framework of finite gap theory of periodic IST^29,30^. The results are also robust to small changes in the numerical values of the parameters related to the carrier wave: changing the numerical value of $\omega _0$ by a 10 $\%$ does not change quantitatively the results obtained by nonlinear spectral analysis. Therefore, the main result obtained within the nonlinear analysis is that the wave packet recorded by the buoy has some nonlinear content, a result that is also comforted by the calculation of the nonlinear length and dispersive length defined as:

5 $$\begin{aligned} L_{nlin}=\frac{\nu }{\gamma }\frac{1}{k_0^3 \langle |A_0|^2\rangle },\;\;\;\; L_{disp}=\frac{\nu ^3}{\kappa }\frac{\omega _0^2}{ k_0}{\Delta T^2}, \end{aligned}$$

where $\Delta T$ is the width of the packet in time which is of the order of 50 s. $\sqrt{\langle |A_0|^2\rangle }$ is a measure of the wave packet amplitude and can be estimated to be 14 m (half the total height). Substituting the numerical values, we obtain $L_{nlin} \sim 7$ km that is shorter than the linear dispersive length $L_{dis} \sim 14.64$ km. We emphasise that the analysis is based on the assumption that the evolution is ruled by the NLS equation at least for short distances.

It is well known that solitons are unstable to transverse perturbations^31,32^ which cannot be avoided in any natural environment; therefore, such structure will eventually disappear. The conservative dynamics of the considered wave packet is influenced by three physical phenomena: the dispersion, the nonlinearity and the diffraction. Based on the 2D+1 NLS equation, the diffraction length can be estimated as:

6 $$\begin{aligned} L_{diff}=\frac{\sqrt{\tanh [k_0 h]}}{k_0} \Delta Y^2, \end{aligned}$$

where $\Delta Y$ corresponds to the width of the wave packet in transverse to the direction of propagation scale. For what concerns the transverse scale, there is no direct measurement; however, to be very conservative, we estimate that the transverse size of the wave packet is at least of 1 km; note that the wavelength in the direction of propagation is almost 600 m. The diffractive effects, due to the finite crest, take place on a scale of 20 km. The nonlinear spatial scale is the shortest one. assuming that the packet is travelling with its group velocity, i.e. $\simeq 56$ km/h, its life time is about of 20 min.

## Discussion

In this paper we have reported on the analysis of a unique time series of surface elevation recorded in the Biscay Bay which shows a giant wave packet whose maximum height is almost 28 m, i.e. one of the largest wave ever measured. Besides its height, the group extends spatially for almost 2 km in the propagation direction. Despite a detailed analysis of the storm that took place during the day of the recording, the condition in which such extraordinary packets are formed is still far from being understood. The analysis reveals that a wind sea is superimposed to a large swell travelling from North-West; the latter appears to be, at least from the analysis of the movement of the buoy, quite unidirectional. The leading order model that can be used to describe locally the dynamics of the wave packet is the Nonlinear Schrödinger equation; we exploit the integrability property of the latter equation and use the IST to establish the nonlinear contents of the wave packet. The analysis, using both PBC and ZBC, reveals that the giant wave packet is characterized by a nonlinear content. We mention that the latter result is based on the assumption that the dynamics is ruled, at least for short time and space, by the NLS equation. Effects of a finite transverse front have to be taken into account, but unfortunately, as most of the times it happens for ocean waves, no spatial measurements are available. A rough estimate of the life-time of the giant wave packet, based on a simple dimensional argument on two-dimensional version of the NLS equation, highlights that the wave packet may have survived at least for 20 min.
